# Accuracy of transition zone in contrast enema to predict intraoperative aganglionosis level in patients with Hirschsprung disease

**DOI:** 10.1186/s13104-020-04945-2

**Published:** 2020-02-25

**Authors:** Zikrul Haikal, Andi Dwihantoro, Hesti Gunarti, Gunadi

**Affiliations:** 1 Pediatric Surgery Division, Department of Surgery, Faculty of Medicine, Public Health and Nursing, Universitas Gadjah Mada/Dr, Sardjito Hospital, Jl. Kesehatan No. 1, Yogyakarta, 55281 Indonesia; 2 Department of Surgery, Faculty of Medicine, University of Mataram, West Nusa Tenggara Mataram, 83126 Indonesia; 3 Department of Radiology, Faculty of Medicine, Public Health and Nursing, Universitas Gadjah Mada/Dr, Sardjito Hospital, Yogyakarta, 55281 Indonesia

**Keywords:** Contrast enema, Aganglionic segment, Hirschsprung disease, Transanal endorectal pull-through, Transition zone

## Abstract

**Objective:**

While frozen section methods have been widely conducted to determine aganglionosis segment during transanal endorectal pull-through (TEPT) for Hirschsprung disease (HSCR) patients in most institutions, some hospitals still rely on contrast enema to predict aganglionosis segments due to unavailability of frozen section facilities. We determined the accuracy of transition zone in contrast enema to predict aganglionosis segments during TEPT. We retrospectively reviewed all contrast enema and frozen sections for HSCR patients under 2 years of age who underwent TEPT at our institution.

**Results:**

We recruited 36 HSCR patients: twenty-six patients (72.2%) had radiographic transition zones limited to rectum, while ten subjects (27.8%) were limited to rectosigmoid. The rectum subgroup of patients showed a concordance of 30.8%, whereas the rectosigmoid subgroup had a concordance of 100%. The sensitivity, specificity, positive predictive value, negative predictive value, and accuracy of contrast enema compared with intraoperative histopathological findings for aganglionosis level were 100% (95% CI 0.60–1.0), 35.7% (95% CI 0.19–0.56), 30.8% (95% CI 0.15–0.52), 100% (95% CI 0.66–1.0), and 50% (95% CI 0.33–0.67), respectively. In conclusions, contrast enema has low accuracy to predict intraoperative aganglionosis segments in HSCR patients, indicating that it might not be utilized to determine aganglionosis level during TEPT.

## Introduction

Hirschsprung disease (HSCR) is a neurodevelopmental disorder characterized by the lack of ganglion cells in the bowel, resulting in functional obstruction in infants [1–3]. Its incidence is higher in Indonesia (3.1:10,000 live births) compared with other populations (1.5, 2.1, and 2.8 cases per 10,000 live births in Caucasians, Africans, and Asians, respectively) [2–4]. It has been shown that the frequency of *RET* rs2435357 and rs2506030 risk alleles is higher in Indonesia than other population [5].

Currently, transanal endorectal pull-through (TEPT) is the most common definitive surgery performed for HSCR patients [6]. While frozen section methods have been widely conducted for intraoperative evaluation to determine the aganglionosis segment during TEPT in most pediatric surgical centers [7–10], there are some hospitals that still rely on contrast enema to predict aganglionosis segment preoperatively due to unavailability of frozen section facilities. Therefore, we aimed to determine the accuracy of transition zone in contrast enema to predict intraoperative aganglionosis segment in HSCR patients who underwent TEPT.

## Main text

### Materials and methods

#### Patients

We retrospectively consecutive identified all contrast enema and intraoperative histopathological evaluations (frozen sections) for HSCR patients under 2 years of age who underwent TEPT at a pediatric surgical center in Indonesia, from January 2016 to December 2017.

Forty-seven HSCR patients were ascertained and 11 cases were excluded due to unavailability of contrast enema because they were performed outside our hospital.

This study was approved by the Institutional Review Board of the Faculty of Medicine, Public Health and Nursing, Universitas Gadjah Mada/Dr. Sardjito Hospital (KE/FK/1255/EC/2018).

#### Intraoperative histopathological findings and contrast enema

**Fig. 1:**
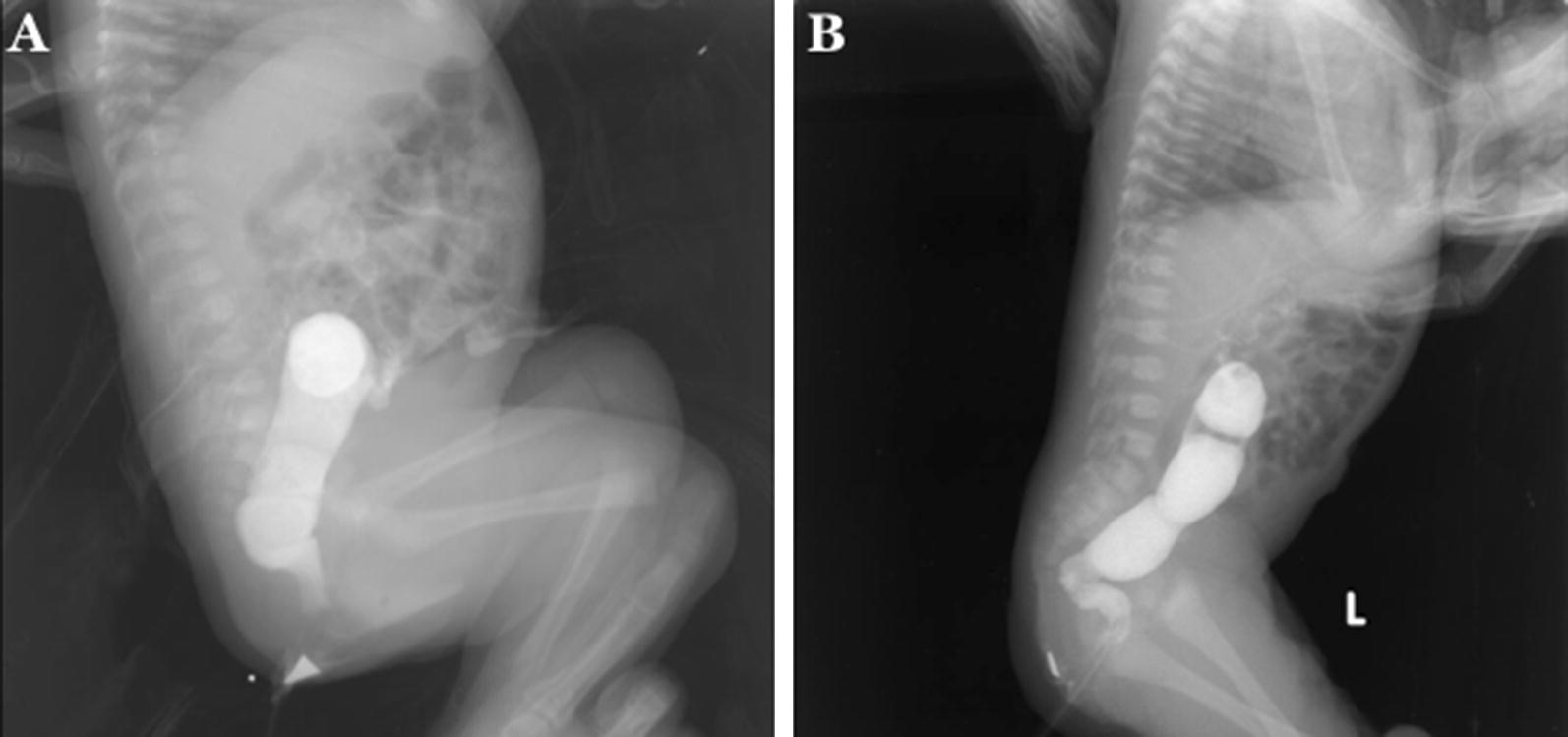
Contrast enema reveals a transition zone in: **a** rectum; and **b** rectosigmoid

For intraoperative histopathological findings, we determined the rectal group if the length of aganglionosis was < 7 cm, and the rectosigmoid group if the length of aganglionosis was ≥ 7 to ≤ 20 cm [8] (Fig. 1). We utilized hematoxylin and eosin (HE) as staining for intraoperative histopathological evaluation [11] (Additional file 1: Fig. S1).

The transition zone on contrast enema was determined as the site of obvious caliber alteration from non-dilated to dilated colon [10].

#### Statistical analysis

Data were presented as number and percentages. The contrast enema transition zone was evaluated for sensitivity, specificity, positive predictive value, negative predictive value, and accuracy. The Cohen’s Kappa index and McNemar test were used to determine the concordance rate between the contrast enema transition zone and the intraoperative histopathological findings.

### Results

We identified 47 patients with HSCR during a 2-year period of study. We excluded 11 HSCR patients because of no contrast enema available, thus, we further analyzed 36 HSCR patients, consisting of 18 males and 18 females. Most of them underwent TEPT at less than 6 months of age (Table 1).

**Table 1:** Clinical characteristics of HSCR patients who underwent TEPT in Dr. Sardjito Hospital, Yogyakarta, Indonesia

Characteristics	N (%)
Gender	
Male	18 (50)
Female	18 (50)
Age at TEPT	
1–6 months old	33 (91.7)
7–12 months old	3 (8.3)
Contrast enema transition zone	
Rectum	26 (72.2)
Rectosigmoid	10 (27.8)

*HSCR* Hirschsprung disease, *TEPT* transanal endorectal pull-through

Twenty-six patients (72.2%) had radiographic transition zones limited to the rectum, while ten subjects (27.8%) were limited to the rectosigmoid (Table 1). The rectum subgroup of patients showed a concordance of only 30.8%, whereas the rectosigmoid subgroup had a concordance of 100% (Table 2).

**Table 2:** Accuracy of contrast enema transition zone to predict intraoperative aganglionosis level in HSCR patients who underwent TEPT

	Contrast enema transition zone		Total
	Rectum	Rectosigmoid	
Intraoperative histopathological findings			
Rectum	8	18	26
Rectosigmoid	0	10	10
Total	8	28	36

*HSCR* Hirschsprung disease, *TEPT* transanal endorectal pull-through

The sensitivity, specificity, positive predictive value, negative predictive value, and accuracy rates of the contrast enema compared with intraoperative histopathological findings for aganglionosis level were 100% (95% CI 0.60–1.0), 35.7% (95% CI 0.19–0.56), 30.8% (95% CI 0.15–0.52), 100% (95% CI 0.66–1.0), and 50% (95% CI 0.33–0.67), respectively (Table 2).

Next, we determined the Cohen’s Kappa index for the contrast enema transition zone and the aganglionosis level of intraoperative histopathological findings, showing its index of 0.198 (slight agreement), while the McNemar test revealed that the sensitivity and specificity rates was significantly different between contrast enema and intraoperative histopathological findings (*p *= 0.0001).

### Discussion

In this retrospective study, we are able to show that the contrast enema transition zone has low accuracy to predict intraoperative histopathological aganglionosis level in HSCR patients who underwent TEPT. Our study found that only 30.8% of contrast studies in the rectal group revealed a transition zone that accurately corresponds with the intraoperative aganglionosis level. Muller et al. [8] reported that the concordance rates in rectosigmoid and rectal subgroups were 57% and 42%, respectively, while another study revealed that the concordance rate of aganglionosis level in the rectosigmoid group was 90% [9].

The accuracy of contrast enema to predict the intraoperative aganglionosis level in this cohort was approximately 50%, lower than previous studies with 62.5% [10] and 89.6% [7]. Moreover, our Cohen’s Kappa index was 0.198, lower than the study by Muller et al. [8] (*vs.* 0.4) and Chen et al. [7] (*vs.* 0.776), but higher than reported by Granero et al. [12] (*vs.* 0.0159). These differences might be due to several reasons, including the type of aganglionosis involved in the study (our study: short-segment *vs.* short- and long-segment [7, 10] *vs.* short-, long- and total colon aganglionosis [8, 9]) and the number of pediatric radiologist who reviewed the transition zone (our study: one *vs.* two pediatric radiologists [10]).

Our findings showed that contrast enema has low concordance with intraoperative histopathological findings (Cohen’s Kappa index = 0.198; *p *= 0.0001), implying that the level of the colonic resection in HSCR should not be based on the contrast enema, it should always be conducted based on the evidence of having ganglion cells on the pulled through segment and to resect the segment with no ganglion cells. For hospitals that do not have frozen section facilities, the pediatric surgeons should look for other strategies, e.g. taking multiple full-thickness biopsies performed on a separate previous surgery.

We classified the contrast enema findings into: (a) the rectal subgroup if the length of aganglionosis was < 7 cm; and (b) the rectosigmoid group if the length of aganglionosis was ≥ 7 to ≤ 20 cm, according to previous study [8], and the fact that the total colon length in children < 2 years old ranges from 52 cm and the average proportional length of rectum is around 4.7–6.2 cm [13].

Moreover, our study did not aim to determine the accuracy of contrast enema for diagnosis of HSCR since all HSCR patients in our hospital were diagnosed using full thickness biopsy samples stained by HE and S100 [11]. Our study’s focus was on prediction of intraoperative aganglionosis level using contrast enema for HSCR patients with short-segment aganglionosis who underwent TEPT, since most HSCR patients are short-segment aganglionosis and currently they often undergo TEPT [2].

### Conclusions

We show that contrast enema has low accuracy to predict intraoperative aganglionosis segments in HSCR patients who underwent TEPT, indicating that it might not be utilized to determine the aganglionosis level during TEPT.

## Limitations

Our report was a retrospective study that might cause a selection bias. Moreover, small number of subjects in our report implies further multicenter study with a larger sample size is necessary to clarify and confirm our findings.

## Supplementary information

**Additional file 1: Figure S1.** Intraoperative histopathological findings using hematoxylin and eosin staining (× 100) show: **a** ganglion cells (arrow); and **b** no ganglion cell.

## Data Availability

All data generated or analyzed during this study are included in the submission. The raw data can be requested from the corresponding author.

## Abbreviations

CI: Confidence interval
HE: Hematoxylin and eosin
HSCR: Hirschsprung disease
TEPT: Transanal endorectal pull-through
