# Supplementary figures and images for: Accuracy of transition zone in contrast enema to predict intraoperative aganglionosis level in patients with Hirschsprung disease
Source: BMC Res Notes. 2020 Feb 25;13:104. doi: 10.1186/s13104-020-04945-2 (PMC7043041; doi:10.1186/s13104-020-04945-2)

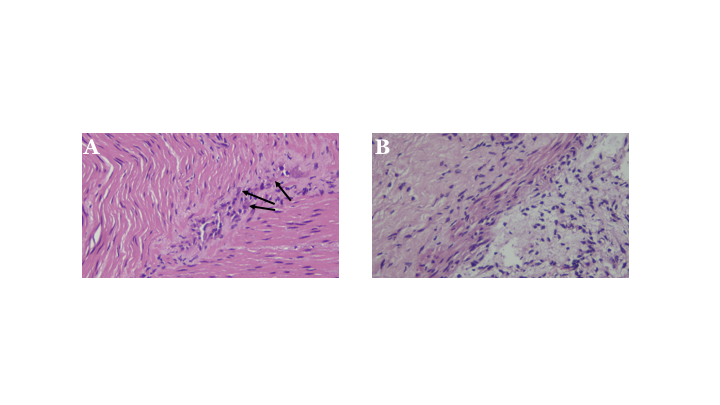

Supplement: Supplementary file 1 — Additional file 1: Figure S1. Intraoperative histopathological findings using hematoxylin and eosin staining (× 100) show: a ganglion cells (arrow); and b no ganglion cell. [file 13104_2020_4945_MOESM1_ESM.tiff]
